# Man’s Underground Best Friend: Domestic Ferrets, Unlike the Wild Forms, Show Evidence of Dog-Like Social-Cognitive Skills

**DOI:** 10.1371/journal.pone.0043267

**Published:** 2012-08-15

**Authors:** Anna Hernádi, Anna Kis, Borbála Turcsán, József Topál

**Affiliations:** 1 Research Centre for Natural Sciences, Institute of Cognitive Neuroscience and Psychology, Hungarian Academy of Sciences, Budapest, Hungary; 2 Department of Ethology, Eötvös University, Budapest, Hungary; 3 HAS-ELTE Comparative Ethology Research Group, Budapest, Hungary; Université Paris 13, France

## Abstract

Recent research has shown that dogs’ possess surprisingly sophisticated human-like social communication skills compared to wolves or chimpanzees. The effects of domestication on the emergence of socio-cognitive skills, however, are still highly debated. One way to investigate this is to compare socialized individuals from closely related domestic and wild species. In the present study we tested domestic ferrets *(Mustela furo)* and compared their performance to a group of wild *Mustela* hybrids and to domestic dogs *(Canis familiaris)*. We found that, in contrast to wild *Mustela* hybrids, both domestic ferrets and dogs tolerated eye-contact for a longer time when facing their owners versus the experimenter and they showed a preference in a two-way choice task towards their owners. Furthermore, domestic ferrets, unlike the wild hybrids, were able to follow human directional gestures (sustained touching; momentary pointing) and could reach the success rate of dogs. Our study provides the first evidence that domestic ferrets, in a certain sense, are more dog-like than their wild counterparts. These findings support the hypothesis that domestic species may share basic socio-cognitive skills that enable them to engage in effectively orchestrated social interactions with humans.

## Introduction

Domestic dogs have long been referred to as “man’s best friend" and not without a reason. Although some would claim that the dog-human relationship is merely a special form of social parasitism [1], many see it as an extremely successful interaction founded on dogs’ human-like social skills [2], [3]. In recent years dogs have become famous for their sophisticated socio-cognitive abilities as it turned out that they are able to follow human momentary distal pointing gestures in order to locate hidden food [3], [4]. To utilize this challenging form of pointing gestures flexibly, dogs must infer something about the communicative-referential meaning of the human’s gestures. Dogs’ high performance in these tasks are surprising because even our nearest primate relatives, the great apes, fail at it [5], [6], as do wolves [5], [7].

Dogs have demonstrated their excellent socio-cognitive abilities in several other tasks as well. Although no differences were found between dogs of blind versus sighted owners in their way of communicating visually about an inaccessible toy object [8] or their sensitivity to human pointing cues [9], dogs are able to take into account the attentional state of humans in a wide range of situations. For example they prefer to beg for food from a human whose eyes are visible [10], [11], and they are less likely to approach forbidden food when a human’s eyes are open than when they are closed [12], (but see [13] for controversial results about dogs and wolves in a similar experiment). Besides being sensitive to the open eyes of a human, dogs also tend to seek eye-contact with the human partner when facing an unsolvable task, contrary to wolves [7]. Face-to-face communication is of great importance for humans [14] and seems to be a crucial aspect of the dogs’ behaviour as well [15].

Many think that these abilities have been formed by the cognitively challenging complex human social environment [5], [16] and, as a consequence of the shared environment, some rudimentary social-cognitive skills such as interspecific attraction and/or sensitivity to human social cues may have developed in some of the domestic species. Through this evolutionary process, the dog as a species has moved from the niche of its ancestor to the human niche [3]. In this new niche dogs have formed a close social relationship with their human partners (e.g. “attachment") [17], and a flexible system for interspecific communication has also emerged [18]. Alternatively or in parallel to these hypotheses, one might expect the socio-cognitive abilities of dogs resulting from their extensive hand rearing and individual socialization to the human environment from a very early age on. One way to find out the role of domestication in the emergence of these special abilities is to study other domesticated species as well.

Although surprisingly little is known about the socio-cognitive abilities of domesticated species other than dogs, the effects of domestication are probably not limited to canids and therefore the comparative exploration of the phenomenon is important. Recent studies found that domestic cats [19], horses [20] and goats [21] are also able to follow human pointing gestures in order to locate hidden food. Furthermore, experimentally domesticated fox kits (selected for tameness for over 45 years) were also found to be more skilled to follow human pointing gestures than fox kits from a control population [22]. These findings indicate that, in line with previous findings on dogs, domestication as a special evolutionary process leads to increased susceptibility to human communication.

Ferrets – a carnivore species of the Mustelidae family originating from wooded and semi-wooded areas [23] – have not yet been experimentally studied in socio-cognitive tasks relating to humans. Although their early history in service of man is obscure, ferrets have probably been domesticated for more than two thousand years [24] by selective breeding from the European polecat *(Mustela putorius)* [25]. Similarly to dogs, ferrets have been bred originally for practical functions (hunting) [26], but nowadays many of them are merely kept as pets (for more details about the history and domestication of *Mustela* see [27]). This makes ferrets an ideal subject to study the effect of domestication on their human related socio-cognitive skills as it seems likely that similarly to dogs (and potentially other domesticated pets), ferrets also adopted to the human niche. Therefore we assumed that in contrast to wild *Mustela* domestic ferrets will show similar behavioural patterns as dogs in socio-cognitive tests. We predicted that both domestic species will show (i) increased tolerance of eye-contact with their owner vs. a stranger, (ii) preference towards their owner as opposed to a stranger when they have to decide from whom to get a piece of food and (iii) utililization of human pointing gestures in order to locate hidden food.

## Results

We tested seventeen domestic ferrets *(Mustela furo)* in three experimental situations where they had to interact either with their owners or with an experimenter and compared their performance to a group of hand-reared wild *Mustela* hybrids (N = 16) and to domestic dogs *(Canis familiaris,* N = 18) (see *Materials and Methods*).

**Figure 1:**
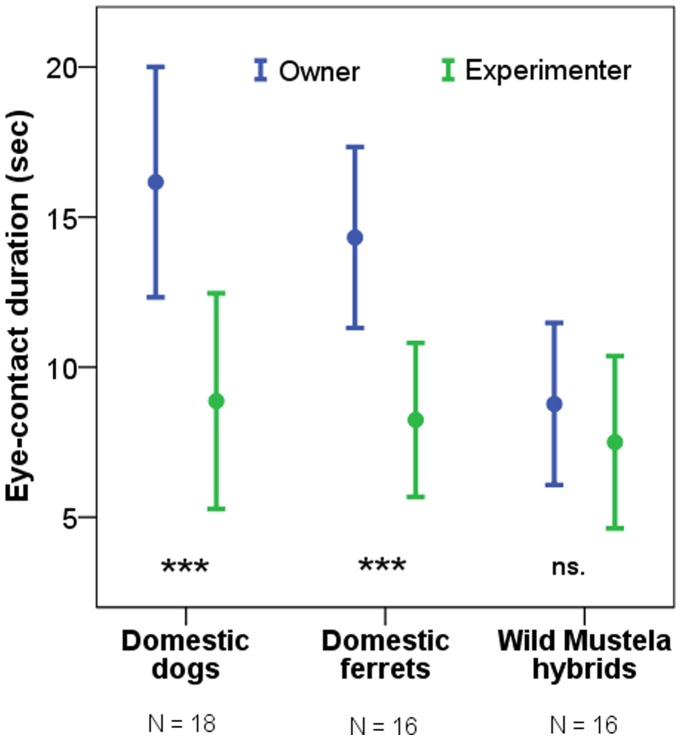
Tolerance of eye-contact. Mean duration of eye-contact during a 30 sec period while the subject was gently held by the owner or the experimenter without restricting head-movements. (***: p<0.001, ns.: p>0.05; error bars represent SD).

First, subjects’ ability to tolerate eye contact was tested both with a familiar (owner) and an unfamiliar (experimenter) human *(Tolerance of eye-contact test).* At the beginning of the trial the human lifted the subject so that it was positioned at his/her face level, established eye contact with it and tried to maintain it’s attention by emitting sounds and/or gently moving the animal during a 30 sec period. Half of the subjects in each group were first tested with the owner and then with the unfamiliar experimenter. This was reversed for the other half of the subjects. We found that both domestic species looked more at the owners’ than at the experimenter’s eyes (paired samples t-test, ferrets: t_(15)_ = 6.088, p<0.001; dogs: t_(17)_ = 6.093, p<0.001), while no such effect was found for the group of wild *Mustela* hybrids (t_(15)_ = 1.092, p = 0.292) (Figure 1). In accordance with this result, the preference for the owner (measured by subtracting the duration of looking at the experimenter’s eyes from the duration of looking at the owner’s eyes) was higher in the domestic ferret group than in wild *Mustela* hybrids (independent samples t-test, t_(30)_ = 3.488, p = 0.001), but no difference was found between domestic ferrets and dogs (t_(32)_ = 0.006, p = 0.995). The above difference between domestic ferrets and wild hybrids resulted from the latter group looking less at the owner’s eyes (t_(30)_ = 3.572, p = 0.001), while no such difference was found between the two groups in case of the unfamiliar experimenter (t_(30)_ = 0.389, p = 0.700). Thus we may conclude that the key difference between domestic ferrets and wild *Mustela* hybrids is the lack of increased tolerance for eye contact with the owner in the latter group.

**Figure 2:**
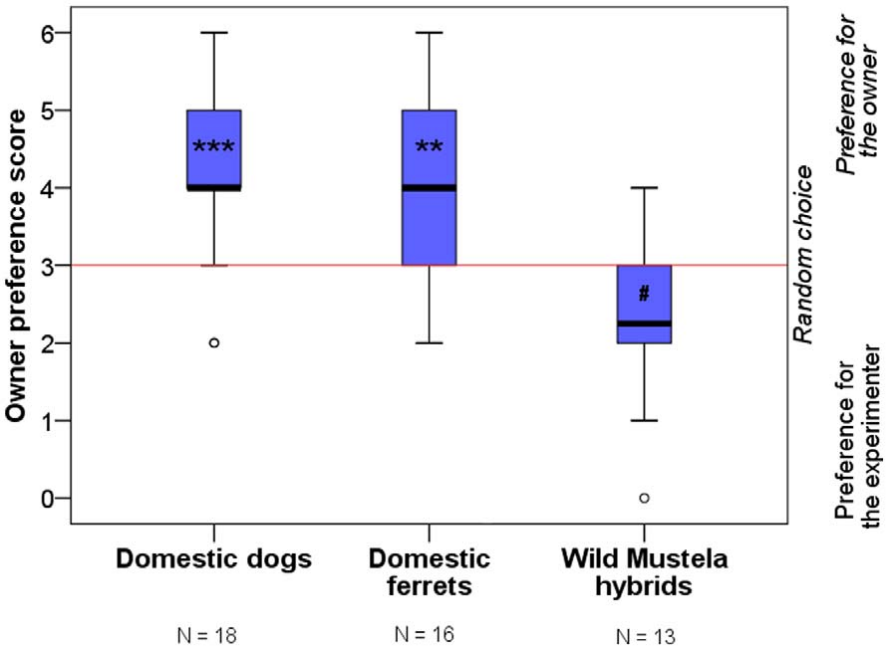
Number of trials with owner versus experimenter preferred out of six in total. Subjects had to choose between their owners and an unfamiliar experimenter while both of them were parallelly holding a piece of food and calling the subject. Red line represents the chance level. (***: p<0.001, **: p<0.01, #: p<0.1; median, quartiles, whiskers and outliers).

Next, subjects had to choose repeatedly (six trials) between a female experimenter and their owner – both of them holding a piece of food – in a two-way choice’ task (*Social-preference test*). Some of the subjects were not willing to participate or completed only part of the trials (see *Materials and Methods* for details), but no difference was found between groups in this respect (Fischer exact test, p>0.1). Both domestic ferrets and dogs chose their owners (as opposed to the experimenter) significantly more often than expected by random selection (Wilcoxon Signed Rank Test; ferrets: T+ = 53.5, p = 0.004; dogs: T+ = 143.0, p<0.001), while the wild *Mustela* hybrid group displayed a marginally significant preference for the unfamiliar experimenter (T− = 38.0, p = 0.074) (Figure 2). Domestic ferrets, in comparison with wild hybrids, selected their owners significantly more often (Mann-Whitney U-Test; U = 19.0, p = 0.001), while no difference was found between the domestic ferrets and dogs (U = 132.5, p = 0.985).

It should be noted that while domestic dogs and ferrets all had female owners, some of the wild *Mustela* hybrids had male owners. No difference was found in the *Tolerance of eye-contact test* between wild *Mustela* with male and female owners in the duration of eye-contact with the owner (N_1_ = 9, N_2_ = 7, t_(14)_ = 0.849, p = 0.409) and with the experimenter (N_1_ = 9, N_2_ = 7, t_(14)_ = 0.262, p = 0.796). However wild *Mustela* with male owners showed higher owner preference in the *Social preference task* than those with female owners (N_1_ = 8, N_2_ = 5, U = 5.1, p = 0.037). This might possibly be explained by the fact that it is easier to make a distinction between a male versus a female, and suggests that wild *Mustela* hybrids were involuntarily tested in an easier version of the *Social preference task*. Interestingly however, despite their ‘advantage’, wild hybrids as a group showed lower preference towards their owners than the two domesticated groups.

Finally we measured the subjects’ responsiveness to two types of human directional gestures (sustained touching and momentary pointing) in two-way object choice tasks (*Responsiveness to human gestures test*). Dogs and ferrets had to choose between two containers – both of them baited with a piece of food – based on the experimenter’s signals (6 touching and 6 pointing trials). In this test wild *Mustela* hybrids were less willing to participate than domestic ferrets both in the *Sustained touching* (Fischer exact test, p = 0.04) and the *Momentary pointing* (Fischer exact test, p = 0.03) task. Furthermore those subjects in the wild *Mustela* hybrid group that did complete all 12 trials had a higher domestic ferret blood ratio (t_(13)_ = 2.12, p = 0.05) than those that did not.

**Figure 3:**
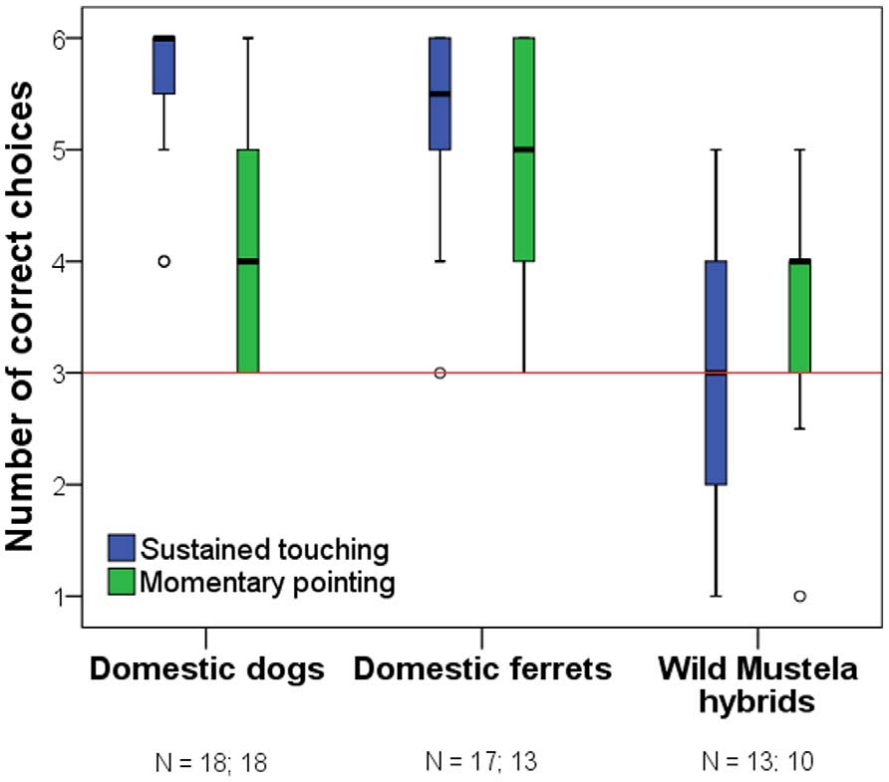
Correct choices out of six trials in the two choice situations based on directional signals. In the sustained touching trials the subject was released while the experimenter was still touching the hiding place. In the momentary pointing trials the experimenter withdraw her hand following the pointing gesture before the subject was released to make a choice. Red line represents chance performance. (***: p<0.001, **: p<0.01, ns.: p>0.05; median, quartiles, whiskers and outliers).

Both domestic ferrets and dogs followed the human directional gestures above chance level in the *Sustained touching* (Wilcoxon Signed Rank Test; ferrets: T+ = 120.0, p<0.001; dogs: T+ = 171.0, p<0.001) and the *Momentary pointing* (ferrets: T+ = 66, p = 0.001; dogs: T+ = 66, p = 0.001) conditions. Wild *Mustela* hybrids however, did not succeed in any of these tasks (touching: T+ = 26.5, p = 0.652; pointing: T+ = 23, p = 0.109) (Figure 3). No effect of the owners’ gender could be observed in case of the wild *Mustela* hybrids *(Sustained touching:* male owner (N = 8): 52.78%, female owner (N = 5): 55.56%, U = 23.5, p = 0.343; *Momentary pointing:* male owner (N = 6): 52.78%, female owner (N = 4): 50.00%, U = 10, p = 0.999). Domestic ferrets outperformed their wild hybrid counterparts in both the *Sustained touching* (Mann-Whitney U-Test; U = 15.5, p<0.001) and the *Momentary pointing* (U = 20.0, p = 0.015) tasks. At the same time no difference was found between the domestic ferrets and dogs in any of the two tasks (*Sustained touching*: U = 114.0, p = 0.231; *Momentary pointing*: U = 68.5, p = 0.584). Furthermore when analyzing only the first trial (it was *a sustained touching trial for all subjects*) both domestic species succeeded in choosing the indicated cup (binomial tests, test proportion: 0.5; ferrets: p = 0.001; dogs: p = 0.008) while wild *Mustela* hybrids did not (p = 1.0).

## Discussion

These findings provide striking evidence that unlike intensively socialized wild *Mustela* hybrids, domestic ferrets and dogs share some social-behavioural characteristics showing the ability to tolerate/prefer eye-contact with their caregivers, displaying preference towards their owners and reliably following human directional gestures. Furthermore it seems that subjects’ willingness to participate (at least in some forms of interactions with humans) is affected by their domestication history. Dogs have already been reported to show specific behaviours towards their owners that manifests for example in increased responsiveness to the owner compared to an unfamiliar human [17], [28]. The specific reaction to separation from and reunion with the human caregiver can also be observed in few months old dog puppies but is seemingly lacking in extensively socialized wolves [29]. Thus similarly to dogs’ other specific social skills [2], [3] one can argue that the ability of showing distinctive behaviour towards the owner also evolved during the process of domestication. Although early socialization might have an important effect on interspecific social relationships, species-specific differences in their social preference towards humans do in fact appear at a very early age in hand raised and intensively socialized dog and wolf puppies [30].

The results obtained from ‘*Tolerance of eye contact*’ and ‘*Social preference*’ tests show striking differences between domestic ferrets and wild *Mustela* hybrids in their specific responsiveness towards their caregivers. Although based on the present results we cannot decide whether the behaviour of ferrets is guided by auditory and/or visual cues, the differences found between domestic and wild individuals allow us to draw a parallel between domestic ferrets and dogs with regard to the studies comparing domestic individuals (ferrets/dogs) with their wild counterparts (*Mustela* hybrids/wolves). Our findings suggest that the owner-preference showed by domestic ferrets is a consequence of their genetic differences from the wild *Mustela* hybrids emerged during their domestication history and that behaving distinctively towards the owner may be a basic trait shared by different domestic species.

It is widely accepted that during the process of domestication dogs were selected for preferring the eye contact with humans and for exploiting this form of social interaction as a potential source of information [19]. Propensity to make eye-contact with humans has already been shown to be an important factor in tasks where subjects have to base their choice on human communicative gestures and accounts for the failure to follow human gestural cues in the case of wolves [7] and apes [31]. The present findings are in line with these arguments as domestic ferrets and dogs – both showing increased tolerance of eye-contact in some sense – were equally successful in following human gestural cues while wild *Mustela* hybrids were not. It is frequently claimed that the utilization of gestural signals presupposes some cognitive skills on the part of the receiver beyond the ability to generalize from everyday communicative interaction with humans to a more controlled experimental situation. However, the fact that domestic ferrets and dogs can rely on human cueing in directing their behaviour in a choice situation is not surprising, since with appropriate human social contact and training, non-domesticated species such as monkeys [32], dolphins [33] and seals [34] are also able to rely on this cue in a two-way object choice test. However, subjects in the present study (contrary to the above mentioned species) did not receive formal training prior to the experiment and were not habituated to the cups containing the food reward. Yet, members of the two domesticated species were successful from their first trial on, whereas members of the wild *Mustela* group were not. This provides evidence of both domestic ferrets and dogs spontaneously relying on human communicative cues and further confirms the assumption that domestication involves genetic changes that lead to enhanced socio-cognitive abilities toward humans. This is in line with previous claims suggesting that relying on human gestural cues may be a basic ability of the domestic species including domestic cats [19], horses [20] and goats [21] that are also able to follow human pointing gestures in order to locate hidden food.

In sum the findings of this study open the door for enlarging the scope of the domestication hypothesis [35]. Besides being the first one investigating human-directed socio-cognitive skills in ferrets, provides an important contribution to the recent debate [36], [37] over whether or not domestication could lead to the emergence of enhanced social abilities. The fact that domestic ferrets seem to be more ‘dog-like’ than ‘wild ferret-like’ regarding their social-affilitative behaviours and responsiveness to human directional gestures strongly supports the notion that (at least some of the) domestic species have acquired a set of social skills that improve their chances to survive in human communities and as a result, they share certain basic capabilities related to social cognition.

## Materials and Methods

### Ethics Statement

No special permission for use of animals in such non-invasive studies is required in Hungary, thus no approval had to be obtained from the local ethics committee for this study. The relevant committee that allows to conduct research without special permissions regarding animals is: University Institutional Animal Care and Use Committee (UIACUC, Eötvös Loránd University, Hungary). Owners volunteered to participate in the project and before the tests an informed verbal consent (which we assumed to be more informative for the participants than a written consent) was received and video recorded. All data was analyzed anonymously.

### Subjects

Three groups of subjects were tested. The first group consisted of 17 privately owned domestic ferrets *(Mustela furo)* (mean age ±SD: 3.6±1.7, 11 males). The second group consisted of 16 privately owned wild *Mustela* × domestic ferret hybrids (wild blood ratio ranged from 1/1–1/16, meaning 0–4 crossbreedings between wild and domestic lines; mean age ±SD: 2.8±2.3, 7 males; 8 European polecat *(Mustela putorius)* hybrids, 4 Steppe polecat *(Mustela eversmanii)* hybrids, 3 European mink *(Mustela lutreola)* hybrids, 1 Siberian weasel *(Mustela sibirica)* hybrid). The third group consisted of 18 adult domestic dogs (*Canis familiaris*) (mean age ±SD: 3.5±2.7, 7 males). Dogs were chosen from small sized breeds (less than 10 kg of weight which were originally bred to hunt and kill vermin (similarly to ferrets) according to their breed standard descriptions (www.fci.be, www.akc.org) (4 Dachshunds, 3 Jack Russell terriers, 3 Chinese naked dogs, 3 Dwarf schnauzers, 3 Yorkshire terriers, 2 West highland white terriers).

Domestic ferrets were all kept in an outdoor enclosure. They entered the house of the owner only occasionally but had daily human contact. Members of the wild *Mustela* hybrid group were either kept in an identical way (N = 6) or lived permanently in the owner’s flat thus having prolonged human contact (N = 10) compared to the domestic ferrets. Keeping conditions for domestic dogs varied from living in a garden without entering the owner’s house to living permanently inside the house, but they all had daily human contact.

### Procedure

Tests were carried out by three female experimenters (AH, AK, BT) with two of them being present at the same time) in a room unfamiliar to the subjects. Domestic ferrets and wild *Mustela* hybrids were tested at their owners’ home in a room that was not familiar to them, while domestic dogs were tested in a room at the Eötvös University. Testing was preceded by a 5-minutes-long habituation period when subjects were allowed to explore the room freely.

Subjects of all groups were engaged in three tests measuring their human-related social behaviours. Some of the subjects had to be excluded due to technical problems (e.g. owner not following the instructions) or because the subject was not willing to participate (see sample sizes indicated on Figures 1, 2, 3). All tests were videotaped for later analysis.

### Tolerance of Eye-contact

Following the habituation period a female experimenter (E1) and the owner (in a counterbalanced order across subjects) made eye-contact with the subjects and was trying to maintain it for 30 seconds. At the beginning of the trial the human lifted the subject so that it was positioned at his/her face level. Both the owner and the experimenter were holding the subjects at the height of their face without restricting head-movements and tried to catch the subjects’ attention by emitting sounds and/or gently moving the subjects. Post-test coding of the videos showed that both the owner and the experimenter spent the same amount of time talking to (domestic ferrets: t_(17)_ = 1.764, p = 0.096; wild *Mustela* hybrids: t_(15)_ = 1.678, p = 0.114; domestic dogs: t_(18)_ = 0.211, p = 0.836) and moving (domestic ferrets: t_(17)_ = 0.826, p = 0.421; wild *Mustela* hybrids: t_(15)_ = 1.742, p = 0.102; domestic dogs: t_(18)_ = 0.031, p = 0.976) the subjects.

We measured the total duration of the subjects looking at the face of the owner and the experimenter respectively with frame-by-frame analysis of the videos. Double coding of 30 videos showed an almost perfect inter-rater agreement (Cohen’s kappa: 0.93). The performance in each group was analyzed by comparing the looking time at the owner versus at the experimenter with paired samples t-tests. The performance of domestic ferrets (the difference between the time looking at the owner and the time looking at the experimenter) was compared to that of wild *Mustela* hybrids and domestic dogs with independent samples t-tests. All statistical tests were two-tailed.

Sixteen domestic ferrets, sixteen wild *Mustela* hybrids and eighteen dogs completed the Tolerance of eye-contact test. 1 ferret was excluded due to technical problems (the owner did not follow the instructions).

### Social-preference Test

After the *Eye-contact* test subjects were engaged in a two way social choice test where they had to choose between a female experimenter (E2) and their owner. Both the experimenter and the owner were crouching 1 m apart from each other, holding a piece of food in their hand. E1 was holding the subject in the middle, 1 m apart from them forming a triangle. First both E2 and the owner simultaneously extended their hand towards the subjects and let them sniff their hands with the food in it while continuously talking. Then E1 released the subject and it could choose between the owner and E2 who were calling it. A choice was coded when the subject approached the hand of the human (owner/experimenter) to a distance of 2 cm or less, with score 1 for choosing the owner and score 0 for choosing the experimenter. The subject received the food from the chosen human but not from the other independently of its choice. If the subject did not approach any of the two humans within a 20 sec period, it was returned to the starting position and received a 0.5 score for that trial. If the subject refused to choose three times in a row, the test was terminated. This choice test was performed six times in total. E2 and the owner changed position (left/right) after each trial and their initial position was counterbalanced among subjects. Subjects that did not make any choice during the test were regarded as “not willing to participate" and were excluded from the analysis of this test (but were included in the other tests). The owner preference score was compared to the 50% chance level (Wilcoxon signed rank test) to analyze the performance in each group. The performance of domestic ferrets was compared to that of wild *Mustela* hybrids and domestic dogs with Mann-Whitney tests. All satistical tests were two-tailed.

Seventeen domestic ferrets, thirteen wild *Mustela* hybrids and eighteen dogs completed the test (with one wild *Mustela* hybrid completing only part of the trials). Three wild *Mustela* hybrids were not willing to participate.

### Responsiveness to Human Gestures Test

#### Sustained touching (6 trials)

Following the social preference test subjects participated in the *Sustained touching* trials (without any pretraining with hiding food in the cups used for this test). E2 placed two cups (both baited with a piece of food) on the floor 1.5 m away from each other and crouched down in between. The owner was holding the subject in the middle 1 m away from E2. The experimenter called the subject’s attention and when it was looking at her, she touched one of the cups. At this point the subject was released and could choose one of the cups while the experimenter was still touching it. Regardless of its choice the subject could eat the food from the chosen cup. A total of six trials were addressed to each subject and the direction of the experimenter’s signal was counterbalanced in RLRLRL or LRLRLR order (for half of the participants the trial sequences were started with leftward touch and for the other half with rightward touch). A choice was coded when the subject ate the food from one of the cups with score 1 for the indicated and score 0 for the non-indicated location. If the subject did not make a choice within 20 seconds, it was led back to the starting point and received a score of 0.5. If the subject refused to choose three times in a row, the test was terminated. Subjects that did not make any choice during the test were regarded as “not willing to participate" and were excluded from the analysis of this test (but were included in the other tests).

Seventeen domestic ferrets, twelve wild *Mustela* hybrids and eighteen dogs completed the *Sustained touching* trials. Four wild *Mustela* hybrids were not willing to participate.

#### Momentary pointing (6 trials)

Following the *Sustained touching* trials subjects received six additional trials with the same setup, but with the experimenter pointing to the cup without touching it (her finger stopped at 5–10 cm away from the cup) and the subject being released only after the withdrawal of the experimenters’ hand.

Thirteen domestic ferrets, ten wild *Mustela* hybrids and eighteen dogs completed the *Momentary pointing* trials (with four wild *Mustela* hybrids completing only part of the trials). Four domestic ferrets and six wild *Mustela* hybrids were not willing to participate.

We recorded the number of correct choices and compared it to the 50% chance level (Wilcoxon signed rank test) for the two types of directional gestures separately. The performance of domestic ferrets was compared to that of wild *Mustela* hybrids and domestic dogs with Mann-Whitney tests. Furthermore performance in the first trial was also examined (binomial test, test proportion: 0.5). All statistical tests were two-tailed.

The video protocol of the tests carried out is available at: http://www.cmdbase.org/web/guest/play/-/videoplayer/51.
